# Functional Organic Materials for Photovoltaics: The Synthesis as a Tool for Managing Properties for Solid State Applications

**DOI:** 10.3390/ma15186333

**Published:** 2022-09-13

**Authors:** Antonio Cardone, Agostina Lina Capodilupo

**Affiliations:** 1 Institute of Chemistry of OrganoMetallic Compounds, Italian National Council of Research, CNR, Via Orabona, 4, 70125 Bari, Italy; 2 Institute of Nanotechnology, Italian National Council of Research, CNR, Campus Ecotekne, Via Lecce-Monteroni, 73100 Lecce, Italy

**Keywords:** energy, organic photovoltaics, functional organic materials, synthetic methodologies, structure-properties correlation

## Abstract

The continuous increase in the global energy demand deeply impacts the environment. Consequently, the research is moving towards more sustainable forms of energy production, storage and saving. Suitable technologies and materials are fundamental to win the challenge towards a greener and more eco-friendly society. Organic π-conjugated materials, including small molecules, oligomers and polymers are a wide and versatile class of functional materials with great potentiality, as they can be used as active matrixes in the fabrication of lightweight, flexible, cheap and large area devices. Their chemical and physical properties, both at a molecular level and mainly in the solid state, are a result of many factors, strictly related to the conjugated structure and functional groups on the backbone, which control the intermolecular forces driving solid state aggregations. The synthesis, through the molecular design, the choice of conjugated backbone and functionalization, represents the first and most powerful tool for finely tuning the chemico-physical properties of organic materials tailored for specific applications. In the present review, we report an overview of our works focused on synthetic methodologies, characterization, structure-properties correlation studies and applications of organic materials designed for energy-involving solid-state applications, organic photovoltaics in particular. The impact of functionalization on electro-optical properties and performance in device are discussed, also in relation to the specific applications.

## 1. Introduction

The energetic demand can be approached both from the production and storage point of view as well as from the energy saving side, and the potential of organic materials can meet both strategies. Organic materials with an extended π-conjugated backbone have semiconducting properties exploitable in a number of applications [1,2], such as organic light emitting diodes [3,4], organic photovoltaics [5,6,7], photocatalysis [8,9], batteries [10,11], and smart applications [12,13]. Due to their great chemical versatility, organic materials can be suitably addressed by synthesis, to be incorporated into various devices for many applications. On the other hand, their chemico-physical properties can be easily tuned by the synthesis, working on molecular conjugated skeleton and functionalization. These structural characteristics, in fact, drive electronic and steric effects, then electro-optical properties and molecular interactions in the solid state, determining the performance of organic materials in device. HOMO and LUMO energy levels, energy gap, charge mobility, thermal and chemical stability, solubility in different solvents, processability, and solid-state aggregation are all properties underlying synthetic control. In this view, clarifying the relationship between chemical structure and the properties of organic semiconductors plays a key role in order to extract their best performance in technological applications. 

Although the molecular conjugated backbone drives the basic electro-optical properties, functionalization deeply affects these properties, and the type of functional group and its position onto the skeleton can drastically change the behaviour of organic semiconductors and then their final performance. In line with these considerations, in this review we present our contribution in the field of organic semiconductor materials for photovoltaic energy, highlighting synthetic methodologies, electrical and optical properties, intermolecular interactions, solid-state organization and performance in device. Organometallic synthetic methodologies were carried out to prepare the organic materials, exploiting their wide versatility, mild reaction conditions and stereoselectivity which allow a rigorous control of the stereochemistry of final compounds. The synthetic methodologies were also selected in order to realize a proper functionalization of the conjugated skeleton, by regarding both the type of functional group and its position on the polymer or molecular skeleton. Electrical, optical and theoretical investigations were performed to deeply understand the effect of chemical structures and functionalization on chemico-physical properties, in order to optimize the performance of the final compound in photovoltaic devices. Then, the performance of synthesized organic materials was investigated by BHJ or DSSC solar cells. Finally, the chemico-physical properties and photovoltaic performance were analyzed and discussed in relation to the chemical structure of materials.

## 2. Results 

### 2.1. Fluorine Impact on Arylenevinylene Materials: Synthesis, Chemico-Physical Properties and Application in BHJ Solar Cells

**Figure 1:**
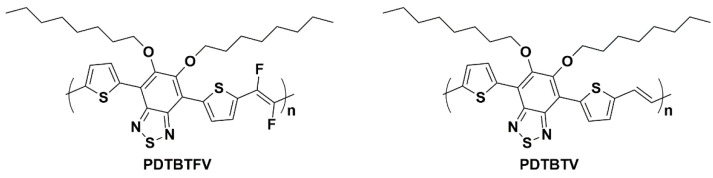
Polymers **PDTBTFV** and **PDTBTV**.

Solar energy is the most abundant and cleanest renewable energy sources available. Organic solar cells (OSCs) as one of the most promising technologies for harvesting solar energy have attracted broad interest thanks to their advantages such as low cost, flexibility, lightweight, semi-transparency, large area, non-toxicity and roll-to-roll large-scale processability. The bulk heterojunction BHJ is a very simple configuration of OSC in which a π-conjugated donor material is blended with an electron acceptor (very often a fullerene derivative) to yield the active layer sandwiched between the electrodes [14,15]. On the way to technological applications, some issues have to be overcome, since critical drawbacks limit the performance of BHJ solar cells, such as efficiency and the low chemical stability of the organic active layer [16,17]. The efficiency of OSCs is attributed to a combination of several features, such as absorption capability, charge-carrier mobility, exciton diffusion length, and the morphology of the active layer plays a key role affecting all these features. To gain good solar cell efficiencies, the electron donor material should possess some characteristics: an extended light absorption matching, as much as possible, the solar spectrum for effective harvesting of the solar photons; high extinction coefficient; HOMO and LUMO energy levels, correctly aligned with those of the acceptor; good hole and exciton transport properties; thermal and chemical stability. So that an ideal donor material blended with PCBM in a BHJ should have a low band gap (between 1.2 and 1.9 eV), with the HOMO energy level in the range −5.2/−5.8 eV and the LUMO energy level in the range −3.8/−4.0, to ensure efficient charge separation and high Voc [18]. Polyarylenevinylenes are a class of materials with low band gap, potentially suitable as donor system in BHJ [19,20]. Following our first results on synthetic methods aimed to prepare arylenevinylene oligomers and polymers with fluorinated double bond [21,22,23,24,25], and on investigations of the effect of fluorination on their chemico-physical properties [26,27,28,29,30], we became interested in studying the impact of the fluorinated vinylene units on the photovoltaic performance of a polyarylenevinylene as donors for BHJ solar cells [31]. For this purpose, we synthesized two soluble conjugated polymers alternating double bonds (either fluorinated or non-fluorinated) with the bis-thienyl-bis-alkoxybenzothiadiazole unit, **PDTBTFV** and **PDTBTV** (Figure 1).

#### 2.1.1. Synthesis of Polyarylenevinylenes for BHJ Solar Cells

**Scheme 1:**
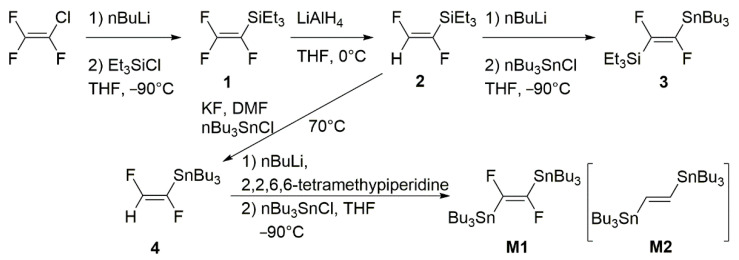
Synthetic route to reagent **M1** and intermediates **1**–**4**.

In order to synthesize arylenevinylene materials with fluorinated and non-fluorinated double bonds, we followed a methodological approach based on the organometallic chemistry, exploiting the Stille reaction, a palladium-catalysed cross-coupling between organo-stannane reagents and organo-halogen derivatives. The Stille reaction is a general and versatile synthetic protocol, which requires very mild reaction conditions, tolerates many functional groups and allows a rigorous stereo-chemical control of the reaction and then of the chemical structure of the final product. The general strategy draws inspiration from the synthetic procedure to prepare the (*E*)-1,2-difluoro-1,2-bis(tributystannyl)ethene **M1**, as reported in Scheme 1.

**Scheme 2:**
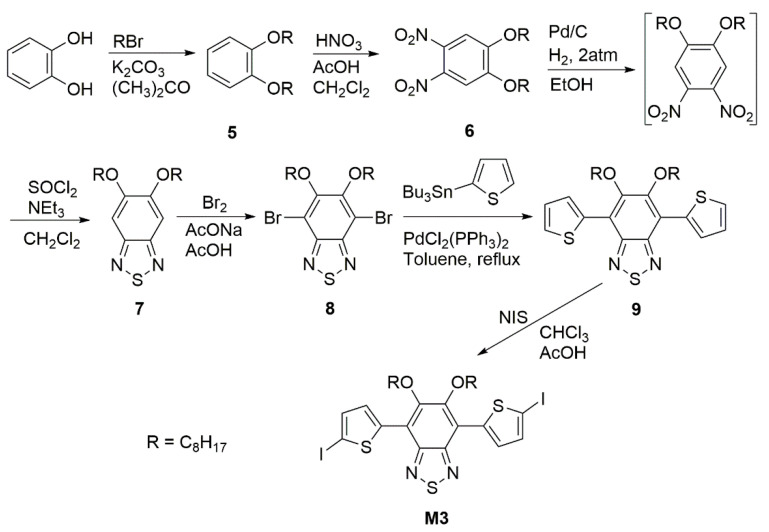
Synthetic route to reagent **M3**.

We adopted a modified literature synthetic procedure, which allows preparing, in addition to the final difluorovinyl-bis-stannane **M1**, useful fluorinated vinyl intermediates (**1**–**4**) which can also be exploited, in turn, as organometallic reagents to prepare arylenevinylene oligomers containing fluorinated double bonds [21]. Compound **M1** is a very useful organo-stannane reagent, suitable to undergo cross-coupling reactions in the Stille conditions, to yield polyarylenevinylenes with *all*-trans fluorinated double bonds. The stereo-regularity of the vinylene units in an arylenevinylene polymer is a key factor, since it determines the effective conjugation length of the backbone and then the chemico-physical and structural properties of the material. Compared to the Stille reaction, traditional synthetic protocols such as the Wittig [32,33,34] reaction, the Knoevenagel [35,36,37,38] condensation, the precursor routes Gilch [39] or Wessling [40] used to prepare arylenevinylene polymers do not allow an accurate control of the stereochemistry of the vinylene units, introducing defects in the conjugated backbone that are detrimental for electrical and optical performance. Materials with semiconductor properties require a high degree of structural regio- and stereo-regularity to exhibit their best performance in a device, so that the Stille reaction, with its high regio- and stereo-selectivity is very attractive as a synthetic methodology to prepare organic semiconducting materials. The analogous of **M1** without fluorine atoms, i.e., (*E*)-1,2-bis(tributylstannyl)ethene **M2**, used to prepare the non-fluorinated polyarylenevinylene, is a well-known reagent, easy to synthesize and exploited in many Stille reactions to prepare a lot of polyarylenevinylenes [25,41,42,43]. As the aryl-dihalide reagent partner of the organostannanes **M1** and **M2**, we used 4,7-bis(5-iodothiophen-2-yl)-5,6-bis(octyloxy)benzo[c][1,2,5]thiazole **M3**, which was synthesized as shown in Scheme 2.

**Scheme 3:**
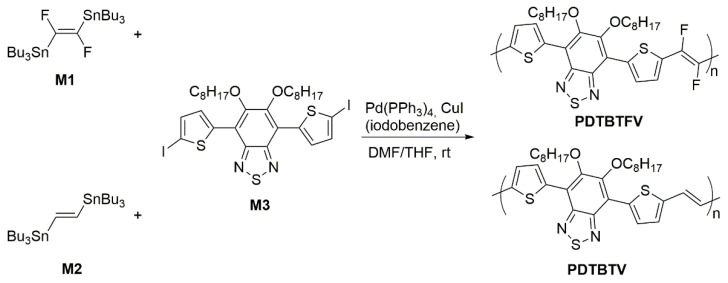
Synthetic route to polymers **PDTBTFV** and **PDTBTV**.

The synthesis of the two polymers, with (**PDTBTFV**) and without (**PDTBTV**) fluorinated vinylene units, was carried out as shown in Scheme 3, via the Stille cross-coupling, using Pd(PPH_3_)_4_ as the catalyst and CuI in stoichiometric amount, in DMF/THF as the solvent, at room temperature, for one week. 

After purification, **PDTBTFV** and **PDTBTV** were obtained as dark-violet and dark-blue powders, respectively, with good yields and good average molecular weights (yield = 75%, Mn = 43,700 and Mn/Mw = 4.46 for **PDTBTFV**, yield = 70%, Mn = 36,900 and Mn/Mw = 4.67 for **PDTBTV**). Both polymers are soluble in chlorinated solvents, such as chloroform, chlorobenzene and *1,2*-dichlorobenzene.

#### 2.1.2. Chemico-Physical Properties and Photovoltaic Performance in BHJ Solar Cells of PDTBTFV and PDTBTV: Impact of Fluoro-Functionalization of Vinylene Units

About the fluoro-functionalization, some papers demonstrated favourable effects of fluorine substitution on the performance of polymers used as donors in blend with PCBM derivatives in BHJ solar cells [44,45,46,47]. Fluorine as a substituent of conjugated materials is appropriate for lowering both the HOMO and LUMO energy levels; this can cause an increase of the open circuit voltage, Voc, in a donor material for BHJ. At the same time, the fluoro-functionalization increase the thermal and chemical stability of organic materials, matching the requirement of organic-based long-lifetime devices. In this direction, the insertion of fluorine atoms on the vinylene unit can be used as a strategic tool to increase the stability of double bonds to the photo-oxidation processes in arylenevinylene materials [28], affecting at the same time electronic properties both in solution [48] and solid state [26,27,28]. In the solid state, in fact, the C-F bonds promote π-stacking interactions [29,49,50,51,52,53]. Experimental and theoretical studies carried out on arylenevinylene polymers and oligomers fluorinated on the vinylene unit highlighted a strong impact of fluorine on the chemico-physical properties of this class of materials, showing a very large blue shift in absorption and emission (with respect to the non-fluorinated homologous), in both solution and solid state. All theoretical studies lead to assign the large blue shift to a remarkable molecular distortion induced by the repulsive interactions between fluorine atoms on the vinylene units and the oxygen atoms of alkoxy groups on the aromatic rings [26,27,29]. Furthermore, all investigations carried out on the arylenevinylenes fluorinated on the double bond highlighted the ability of fluorine, as the substituent, to improve the stability of these materials against the photo-oxidation processes, as well as to deeply affect the electronic properties, disclosing it as one of the powerful tools to obtain blue emission from this class of materials, both in solution and solid state [28]. 

Polymers **PDTBTFV** and **PDTBTV** were used as donor materials blended with PCBM in BHJ solar cells and the photovoltaic performance were recorded on non-optimized devices [31]. Different donor/acceptor (polymer/PCBM) weight ratios were tested and the results of cell performance are reported in Table 1. 

Cell performance data shows the superiority of the fluorinated polymer **PDTBTFV** as a donor over the non-fluorinated **PDTBTV**, with higher values of Voc, FF and then η. For both polymers, the thermal annealing of the blend in the range 70–150 °C substantially does not affect the photovoltaic performance, according to the data of the differential scanning calorimetry (DSC), which shows the lack of any transition. 

**Figure 2:**
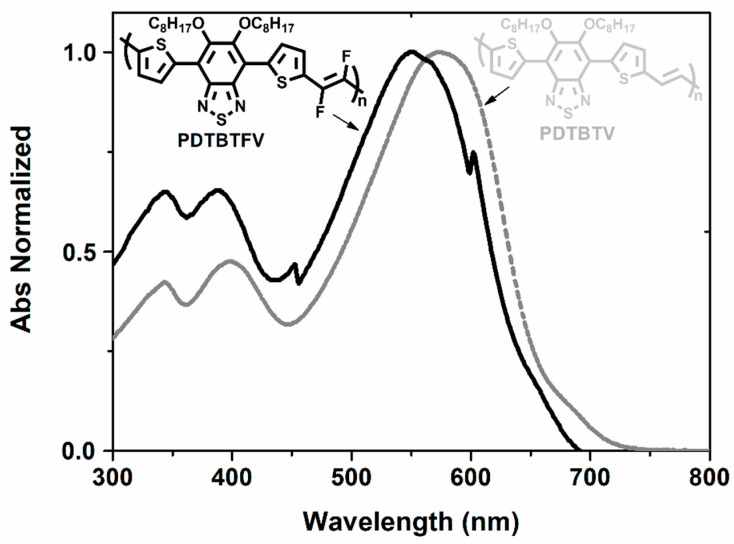
Absorption spectra (normalized) in CHCl_3_ solution of polymers **PDTBTV** and **PDTBTFV**.

In order to shed light on the results achieved regarding the capability of fluorine to enhance the photovoltaic properties of the arylenevinylene structure investigated, we carried out theoretical, electrochemical and optical characterization. The absorption spectra of **PDTBTFV** and **PDTBTV** recorded in a chloroform solution are shown in Figure 2.

**Figure 3:**
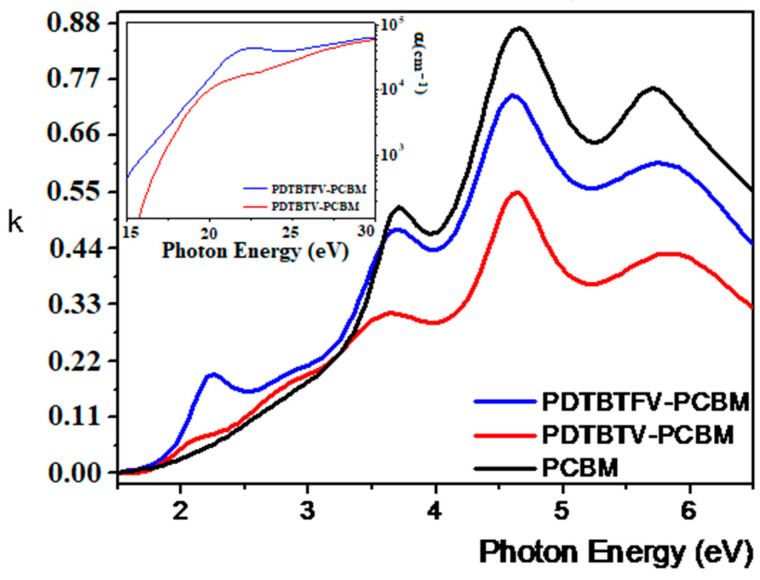
Ellipsometric spectra of the extinction coefficient, and in the inset, of the absorption coefficient for the fluorinated **PDTBTFV** and non-fluorinated **PDTBTV** polymers blended with PCBM (polymer/PCBM ratio 1:1 for both blends). For comparison, the spectrum of PCBM is also shown. Reprinted (adapted) with permission from ref. [31]. Copyright {2014} The Royal Society of Chemistry.

Both polymers absorb in a wide range of the visible region, with maxima picked at 548 nm (2.26 eV) for **PDTBTFV** and 569 nm (2.18 eV) for **PDTBTV**, with a blue-shift of about 21 nm (0.08 eV) induced by fluorination of vinylene units. According with the results of the TD-DFT calculations, the blue-shift is a result of the mesomeric and inductive effects of fluorine atoms. In the solid state, ellipsometry investigations showed the same trend in the absorption spectrum observed in solution, with an optical band gap of the films of polymers found at 1.79 eV for **PDTBTFV** and 1.69 eV for **PDTBTV**. The HOMO and LUMO energy levels were investigate by cyclic voltammetry, and based on the Koopmans’ theorem, they were estimated to be HOMO −5.20 eV for **PDTBTFV** and −5.01 for **PDTBTV**, LUMO −3.14 for **PDTBTFV** and −3.03 for **PDTBTV**. These data reveal an overall stabilization of both HOMO and LUMO energy levels induced by fluorination of vinylene units, in agreement with the results of DFT calculations. The ellipsometric investigations were very interesting, revealing a remarkable effect of fluoro-functionalization of the vinylene units inducing a significant enhancement of thin film light absorption in **PDTBTFV** with respect to **PDTBTV**. This result can be attributed to a different packing of the polymer chains in the solid state, with the fluorination of the vinylene units being responsible for the increased intermolecular interactions by π-stacking interactions and then for the observed increased absorption coefficient. Moreover, ellipsometry analysis carried out on the blend of polymers and PCBM used for the BHJ solar cells revealed that the absorption coefficient of the **PDTBTFV**-PCBM blend still remains higher than that of the **PDTBTV**-PCBM blend in the region of the main optical transition of the polymers (Figure 3). 

The higher capability of light absorption of the fluorinated polymer **PDTBTFV** might be a main reason for its better photovoltaic performance with respect to the non-fluorinated **PDTBTV**, since a higher light absorption has a greater chance of generating more charge carriers in the active blend. In summary, two main synergetic effects can be considered the basis of the favourable impact of fluorination of vinylene units on the photovoltaic performance of the polyarylenevinylene structure investigated: (a) the lowering of HOMO and LUMO energy levels, leading to an increase in Voc; (b) the increase in the molar extinction coefficient. The latter is very attractive in the perspective of reduction of film thickness in BHJ solar cells, which is necessary to enhance the charge carrier generation at the interface and enable the collection of carriers at the electrodes against their tendency to recombine.

### 2.2. Small Molecules for Dye-Sensitized Solar Cells DSSCs: From Synthesis to Photovoltaic Performance in Device

In the framework of the new generation of photovoltaics which can provide an alternative for the exploitation of solar energy, dye-sensitized solar cells (DSSCs) are hybrid photovoltaic devices with great potentiality, being inexpensive and environmentally friendly. Starting from the pioneering work of O’Regan and Grätzel in 1991 [54], they have been attracting a lot of interest as technically and economically credible alternative to the p-n junction solar cells. A DSSC is substantially a photoelectrochemical solar cells consisting of an organic dye sensitizing a mesoporous TiO_2_ working electrode (WE), a redox electrolyte and a counter electrode (CE). Since both the WE and CE can be semi-transparent, both sides of the solar cell can be exploited for illumination. The efficiency of a DSSC is strictly connected with the nature of organic dye, WE, CE and electrolyte, and the interactions between all these components. The organic dye plays a key role in the performance and stability of the device, and the improvement of its performance is fundamental to gain advances in cell efficiency. Since high extinction coefficients are needed to gain high photovoltaic efficiencies, pure organic dyes are preferred with respect to traditional and first used metal-organic complexes [55,56,57,58]. Organic dyes, in fact, have unique advantages such as being low cost, non-toxic, having a high extinction coefficient, a flexible molecular design, easy tunability of molecular structure and additionally of optical and electrochemical properties [59,60,61,62]. Despite these attractive features, drawbacks such as a low open circuit voltage and high recombination losses limit the efficiencies in the final device [63,64,65]. An ideal organic dye should possess features such as a high efficiency in device, chemical and thermal stability, and an easy and low cost of the synthetic procedure used for its preparation. The best performing organic dyes have a donor-π-bridge-acceptor (D-π-A) structural architecture, where A is mainly the cyanoacrylic group [66,67,68,69,70,71], while D and π are the units on which most of the efforts have been focused, in order to gain improvements of photovoltaic performances. As donor systems, the aryl amines resulted to be the best performing, and the research efforts have been devoted to the strengthening of the electron-donating power and to the introduction of bulky chains preventing the aggregation and recombination processes on the TiO_2_. However, the π-bridge, linking the donor and acceptor units, plays a crucial role in the whole performance of the organic dye, by adjusting the HOMO and LUMO energy levels and extending the absorption range [60]. Moreover, since the π-bridge has to favour the electron migration from the donor to the acceptor, a planar geometry (fused rings) should be preferred. In fact, the literature reports, in general, high performances for organic dyes containing a fused ring as the π-bridge [72,73,74,75,76,77,78]. 

#### 2.2.1. Benzothienobenzothiophene (BTBT)-Based Organic Dyes

**Figure 4:**
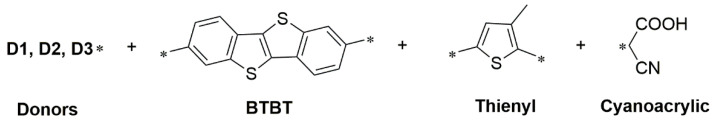
Units to be reacted to prepare the organic dyes.

In the framework of our interest in organic dyes for DSSCs, we focused our attention on a D-π-A molecular architecture based on an intriguing fused-ring planar system as the π-bridge, namely the [1]benzothieno[3,2-b]benzothiophene (BTBT). BTBT has been largely investigated for its excellent charge mobility in field-effect transistors [79,80,81], but not as a component of materials for photovoltaic applications. The charge-transport properties and the very easy and cheap synthetic access suitable to large scale production of BTBT make this molecular unit very attractive for photovoltaic purposes, especially in DSSC devices. We selected the cyanoacrylic group as the acceptor unit A, which was linked to the BTBT unit by the 3-methylthiophene ring (see Figure 4 for the units to connect for the synthesis of organic dyes).

As donor D we selected three electron-donor groups, characterized by a different donating power and long alkyl chains (octyl chains) to prevent aggregation processes [82].

##### Synthesis of BTBT-Based Organic Dyes

**Scheme 4:**
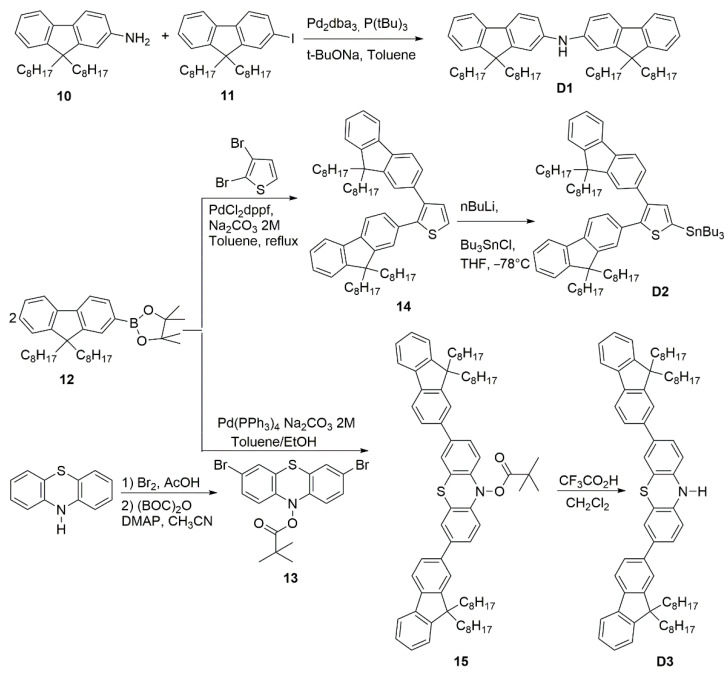
Synthetic route to intermediates containing the donor unit **D1**, **D2** and **D3**.

The synthetic strategy carried out to prepare the three intermediates containing the donor unit **D1**, **D2** and **D3** is shown in Scheme 4.

**D1** was synthesized by reacting the fluorene derivatives **10** and **11** in the Buchwald-Hartwig reaction conditions. **D2** was synthesized by reacting fluorenyl-boronate **12** with 2,3-dibromothiophene, in the Suzuki-Miyaura reaction conditions. Following the intermediate **14** was converted in the stannyl derivative **D2** by lithiation and quenching with tributylstannylchloride. **D3** was synthesized by reacting **12** with the phenothiazine derivative **13** in the Suzuki-Miyaura reaction conditions and following deprotection of the N-boc group.

**Scheme 5:**
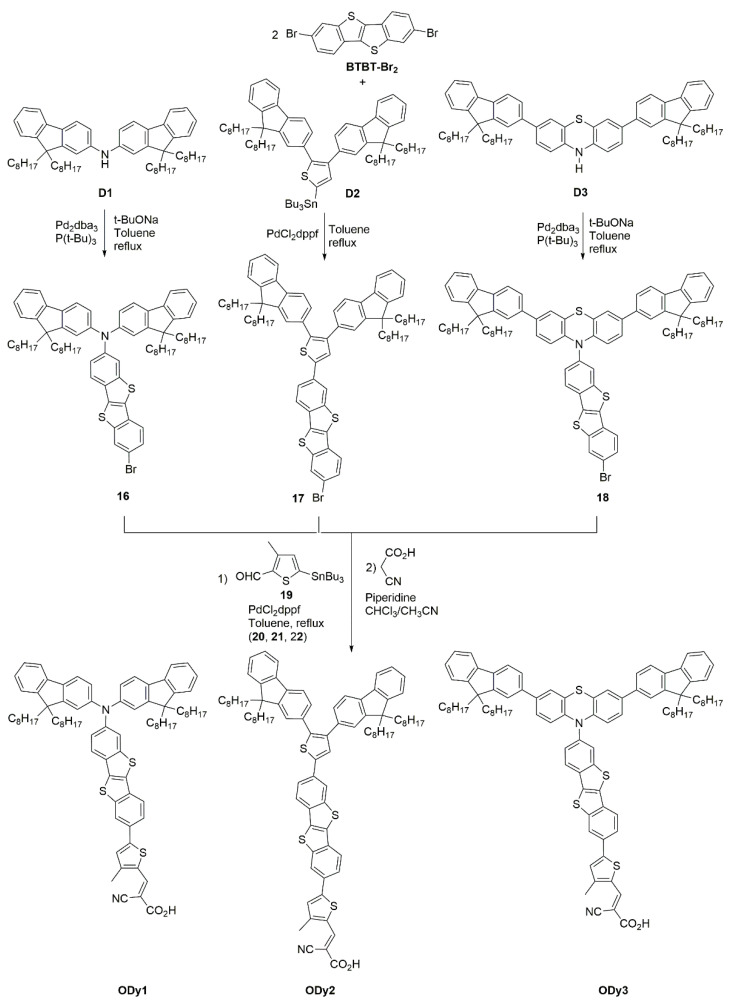
Synthetic route to organic dyes **ODy1**, **ODy2** and **ODy3**.

The synthetic strategy carried out to prepare the organic dyes **ODy1**, **ODy2** and **ODy3** is detailed in Scheme 5.

In the first step, the dibromo-[1]benzothieno[3,2-b]benzothiophene (**BTBT-Br**₂) derivative was reacted with the three intermediates **D1**, **D2** and **D3** to yield the intermediate compounds **16**, **17**, **18**. **BTBT-Br₂** was coupled with **D2** in the Stille reaction conditions, and was coupled with **D1** and **D3** in the Buchwald-Hartwig reaction conditions. The resulting intermediates **16**, **17** and **18** were submitted to the Stille cross-coupling with the stannylthiophene derivative **19**, to yield compounds **20**, **21** and **22**. Finally, compounds **20**, **21** and **22** were reacted with cyanoacrylic acid to yield the final organic dyes **ODy1**, **ODy2** and **ODy3**, which were obtained as red-orange, red and light-red powders, respectively.

##### Optical and Electrochemical Properties, Photovoltaic Performance of BTBT-Based Organic Dyes

**Figure 5:**
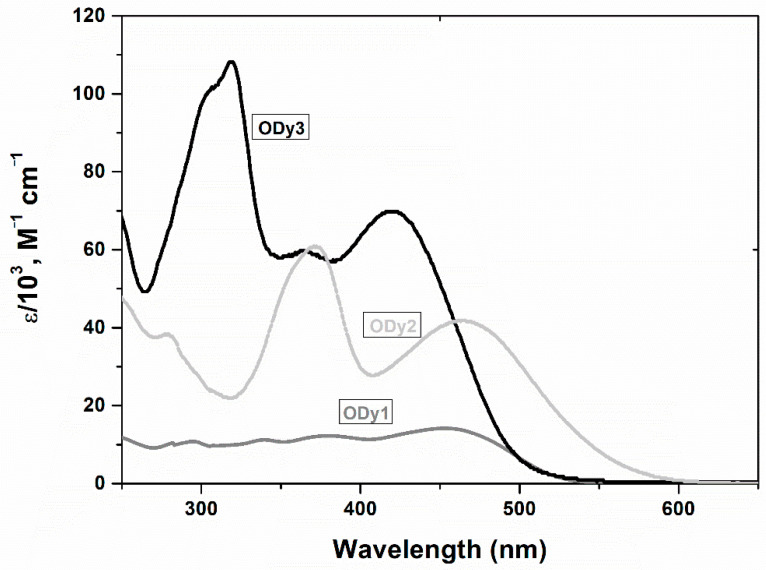
Molar absorption spectra of organic dyes **ODy1**, **ODy2** and **ODy3** in CHCl₃. Reprinted (adapted) with permission from ref. [82]. Copyright {2016} American Chemical Society.

Optical properties of the synthesized organic dyes **ODy1**, **ODy2** and **ODy3** were investigated by recording molar absorption spectra in chloroform solution (Figure 5).

All dyes show broad absorption spectra: **ODy1** in the range 250–500 nm, with the maximum picked at 453 nm and an extinction coefficient of 14,190; **ODy2** in the range 300–550 nm, with two main bands picked at 372 and 464 nm, and extinction coefficients over 60,000 and 42,000, respectively; **ODy3** in the range 250–500 nm, with two main bands picked at 320 and 420 nm, and extinction coefficients over 100,000 and 69,000, respectively. As evident, the introduction of stronger electron-donor arylamine units in **ODy2** and **ODy3**, with respect to the bis-fluorenylthiophene donor in **ODy1**, strongly impacts the absorption capability of the dyes with a strong enhancement of the extinction coefficient. The increase in the absorption capability leads to an enhancement of photovoltaic performance (as reported below). Moreover, the introduction of a more complex donor system, based on the phenothiazine unit in **ODy3**, further increases the absorption capability of the related dye, beside a blue shift of the absorption spectrum. Nevertheless, the photovoltaic performance of **ODy3** does not follow the strong improvement of the extinction coefficient, due to the blue shift of the absorption spectrum.

Cyclic voltammetry CV was performed to investigate the redox behaviour of the dyes and the HOMO and LUMO levels, together the energy gap Eg were estimated, as reported in Table 2.

The HOMO energy levels of the dyes are more positive than the redox potential of I^−^/I_3_^−^ (0.4 V vs. NHE) [83], therefore the oxidized dyes obtained after electron injection into the conduction band of TiO₂ could be regenerated by the reducing species in the electrolyte solution. The LUMO energy levels of the dyes are sufficiently more negative than the conduction band edge of TiO₂ (−0.5 V vs. NHE), therefore there is a sufficient driving force for electron injection from the excited dyes to the conduction band of TiO₂ [84]. These electrochemical data suggest these organic dyes could be suitable as sensitizer in DSSCs.

Since the optical and electrochemical properties of the organic dyes **ODy1, ODy2** and **ODy3** resulted suitable for photovoltaic applications, we tested them in DSSC solar cells and the results are shown in Table 3.

Taking in account the high planarity of the BTBT system, which in turn favours the planarity of the dyes, unfavourable π−π stacking and fast intramolecular charge transfer should be expected, which may lead to intermolecular quenching or back transfer of the injected electrons from the TiO₂ conduction band. In these cases, the addiction of chenodoxycholic acid (CDCA) to the solution containing the dyes could be very useful. CDCA competing with the dye in binding the titania surface, can insert itself between the molecules of sensitizer, preventing or minimizing the detrimental aggregation effects [85,86,87]. In addition, the introduction of coadsorbents such as CDAC allows for a more uniform coverage of the inorganic semiconductor surface, hence lowering the probability of recombination processes between electrons injected and I_3_^−^ or other acceptor species. Actually, the introduction of the coadsorbent CDCA favourably impacted on the photovoltaic performance, with an increase in Voc and Isc parameters and then of efficiencies.

The organic dyes **ODy1, ODy2** and **ODy3** based on the BTBT π-bridge showed good photovoltaic parameters, with efficiencies in the range 4–6%. Although these values are not as high as other optimized structures [72,73,74,77,88] based on fused ring systems, they are comparable [76,89,90,91,92] and sometimes competitive [67,93,94,95,96,97] with other dyes. These achievements support the choice of the BTBT planar system as π-bridge with good potentiality in organic dyes for DSSCs.

#### 2.2.2. Dibenzofulvene (DBF)-Based Organic Materials for Dye-Sensitized Solar Cells DSSCs: Synthesis, Structure-Properties Correlation and Cell Performance

**Figure 6:**
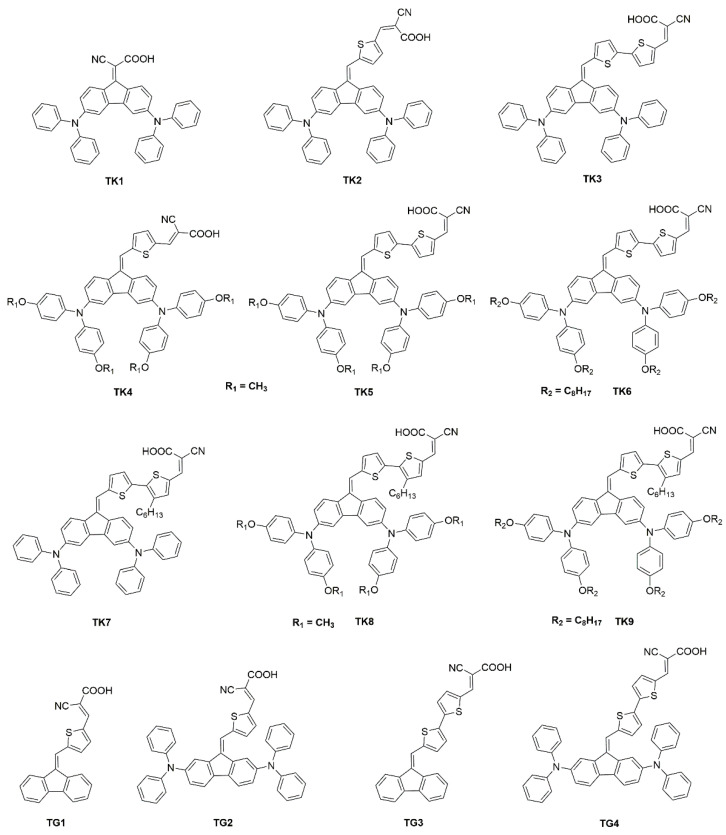
Dibenzofulvene (DBF)-based organic dyes.

In the framework of our interest in organic small molecules for DSSC solar cells, especially for new π-bridges in D-π-A structures, we devoted our attention on the dibenzofulvene (DBF) system, having a planar configuration and whose chemical structure allows to connect two donor units per DBF molecule in a new structure of 2D-π-A type. In such a dye structure, two electron-donating units can push electrons to the electron-acceptor anchoring group. The DBF unit has been used as π-conjugated system in advanced functional materials for optoelectronic devices, such as organic field effect transistors (OFETs) [98], organic light emitting diodes (OLEDs) [99] and organic photovoltaics [100,101,102], but until our work, to the best of our knowledge, was lacking a systematic and in-depth investigation of its potential as a component of dyes for DSSC solar cells. By exploiting the electron delocalization along the ylidene system, the electron-donating effect of donor groups linked to the DBF skeleton can be directed to the acceptor group, thus allowing the electron injection into the TiO_2_ component of the cell. The position of donor groups onto the DBF skeleton, as well as their electron donating power can be exploited as chemical tools to tune the electronic properties of the organic dyes, and at the whole, their performance in cell. We selected diarylamines as donor groups, the cyanoacrylic acid as the acceptor group and the π-bridges consisting of DBF alone or DBF connected to thiophene or bithiophene systems [75,103,104,105]. The structures of the synthesized dyes are shown in Figure 6.

##### Synthesis of Dibenzofulvene (DBF)-Based Organic Dyes

**Scheme 6:**
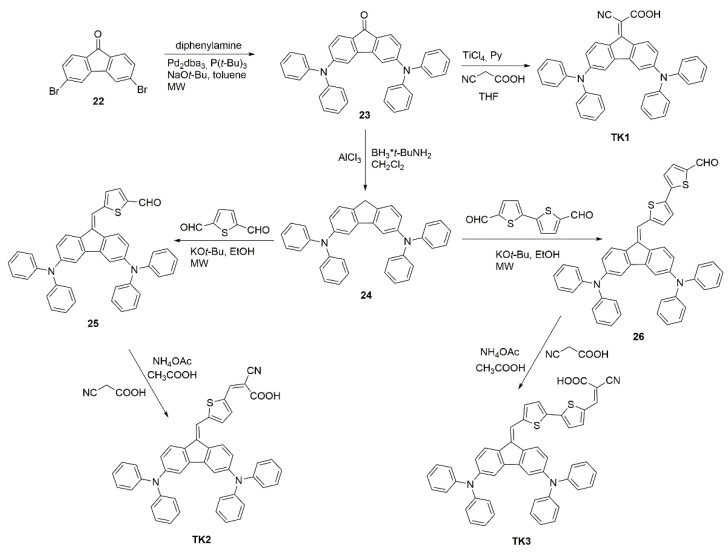
Synthetic route to dyes **TK1, TK2** and **TK3**.

Organic dyes **TK1**, **TK2** and **TK3** were synthesized as reported in Scheme 6 [75].

To yield the intermediate compound **23,** 3,6-dibromofluorenone **22** was reacted with diphenylamine in microwave-assisted Buchwald-Hartwig reaction conditions. Compound **23**, when reacted with cyanoacrylic acid in the presence of TiCl_4_ and pyridine yields the dye **TK1**, when submitted to the reduction of the carbonyl group by tert-butylamine-borane complex and aluminum chloride yields the intermediate **24**. Compound **24** was reacted with thiophene-2,5-dicarbaldehyde and 2,2′-bithiophene-5,5′-dicarbaldehyde in a microwave reactor to yield intermediates **25** and **26**, respectively. Finally, compounds **25** and **26** reacted with cyanoacrylic acid yield the dyes **TK2** and **TK3**, respectively. The dyes **TK1**, **TK2** and **TK3** differ for the π-bridge, which is the DBF unit alone in **TK1**, the DBF-thiophene unit in **TK2** and the DBF-bithiophene unit in **TK3**.

**Scheme 7:**
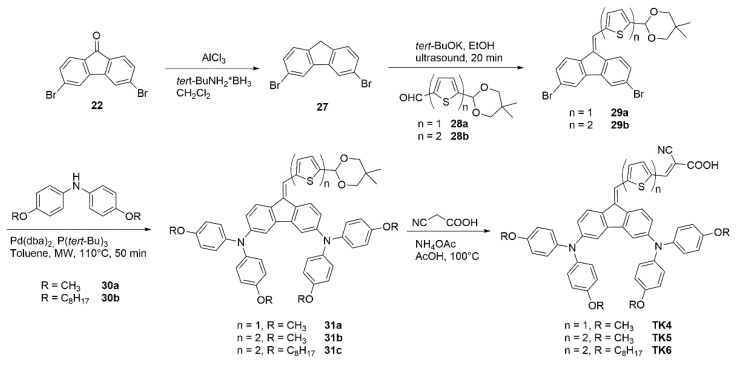
Synthetic route to dyes **TK4, TK5** and **TK6**.

Organic dyes **TK4**, **TK5** and **TK6** were synthesized as reported in Scheme 7 [104].

Compound **22** was submitted to the reduction of the carbonyl group to yield intermediate **27**, which in turn was reacted with compounds **28a** and **28b** to yield intermediates **29a** and **29b**, respectively (see reference [104] for details about the synthesis of compounds **28a**, **28b**, **30a** and **30b**). Compounds **29a** and **29b** were then reacted with **30** in the microwave-assisted Buchwald-Hartwig reaction conditions, to yield the intermediates **31a**, **31b** and **31c**, respectively. Finally, **31a**, **31b** and **31c** were reacted with cyanoacrylic acid to yield the organic dyes **TK4, TK5** and **TK6**.

**Scheme 8:**
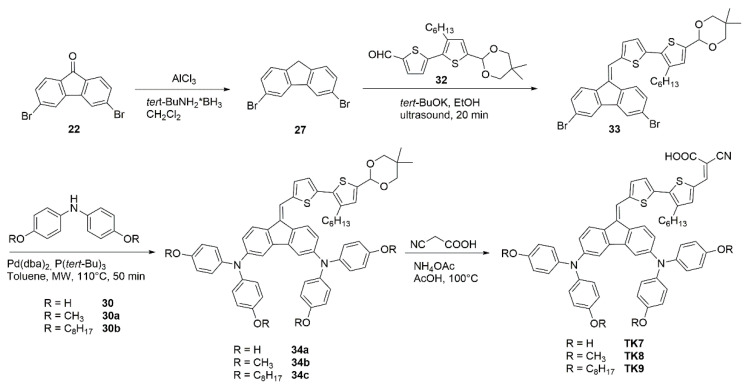
Synthetic route to dyes **TK7, TK8** and **TK9**.

Organic dyes **TK7, TK8** and **TK9** were synthesized as reported in Scheme 8 [103].

For details about the synthesis of intermediates **30a, 30b** and **32** see reference [103]. Compound **22** was submitted to the reduction of the carbonyl group to yield intermediate **27**, which was reacted with **32** to yield intermediate **33**. Following, intermediate **33** was reacted with intermediates **30**, **30a**–**b** in the microwave-assisted Buchwald-Hartwig reaction conditions, to yield intermediates **34a**–**c**. Finally, intermediates **34a**–**c** were reacted with cyanoacrylic acid to yield the organic dyes **TK7**, **TK8** and **TK9**.

**Scheme 9:**
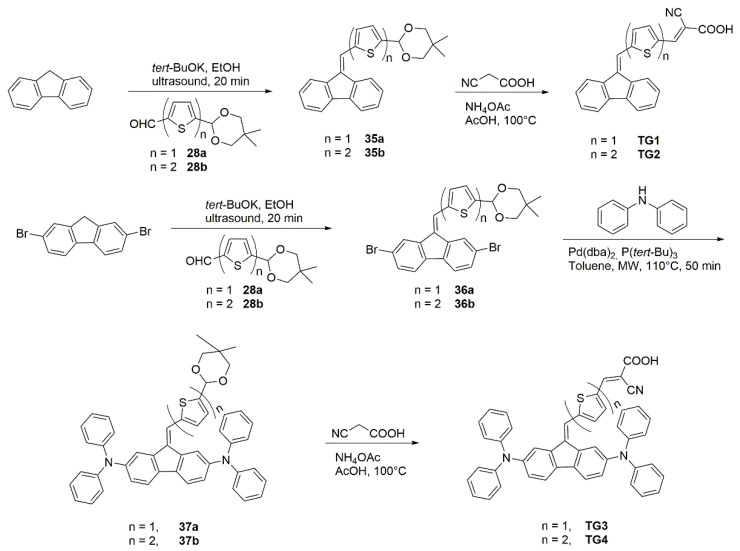
Synthetic route to dyes **TG1**, **TG2**, **TG3** and **TG4**.

Organic dyes **TG1, TG2, TG3** and **TG4** were synthesized as reported in Scheme 9 [105].

Fluorene was reacted with intermediates **28a** and **28b** (see reference [105] for details about their synthesis) to yield intermediates **35a** and **35b**, which in turn were reacted with cyanoacrylic acid to yield the organic dyes **TG1** and **TG2**. 2,7-dibromofluorene was reacted with intermediates **28a** and **28b** to yield intermediates **36a** and **36b**, which in turn were reacted with diphenylamine, in the microwave-assisted Buchwald-Hartwig reaction conditions, to yield intermediates **37a** and **37b**. Finally, compounds **37a** and **37b** were reacted with cyanoacrylic acid to yield the organic dyes **TG3** and **TG4**.

##### Electro-Optical Properties, Structure-Properties Correlation and Photovoltaic Performance of Dibenzofulvene (DBF)-Based Organic Dyes

**Figure 7:**
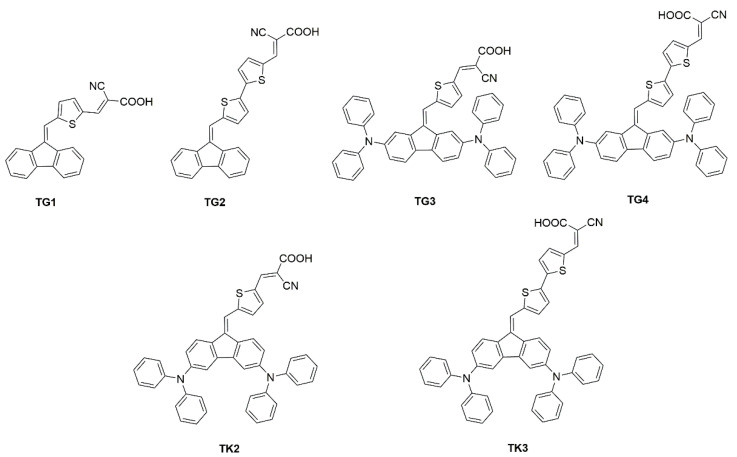
Dibenzofulvene (DBF)-based organic-dyes.

In view of design and synthesis of DBF-based organic dyes with structure 2D-π-A for DSSC, the DBF molecule is a versatile chemical structure which can be easy functionalized in the 2,7- and 3,6-positions by electron-donor groups. This allows for the design of two different classes of dyes, with different structural and chemico-physical behaviour, in which a different electronic conjugation is expected between the donor and the acceptor units, the last being connected to the donor by the ylidene system and the π-spacer of the π-bridge. In line with these assessments, we studied the effects of donor position on DBF-based organic dyes for DSSC [105], starting our investigation from structurally simply dyes, shown in Figure 7.

**Figure 8:**
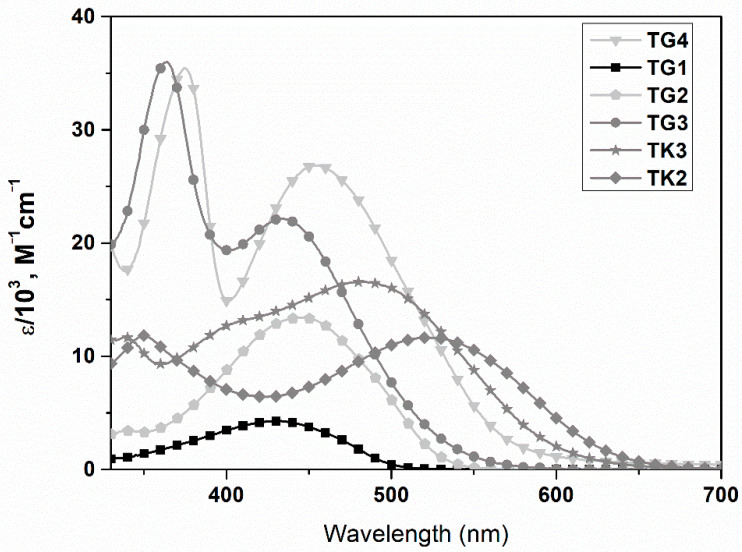
Molar absorption spectra of dyes **TG1, TG2, TG3, TG4, TK2** and **TK3** in CH₂Cl₂ solution. Reprinted (adapted) with permission from ref. [105]. Copyright {2017} Clearance Center’s RightsLink of Spring Nature.

**TG1** and **TG2** are two basic structures without donor groups on the DBF unit, and containing one or two thiophene rings, respectively, as spacer between the ylidene system and the cyanoacrylic acceptor unit. **TG3** and **TG4** can be considered as deriving from **TG1** and **TG2**, respectively, with the introduction of the diphenylamine donor unit in the 2,7-positions of the DBF structure. **TK2** and **TK3**, instead, can be considered as deriving from **TG1** and **TG2**, respectively, with the introduction of diphenylamine donor unit in the 3,6-positions of DBF structure. The absorption spectra of dyes recorded in CH₂Cl₂ are reported in Figure 8 as molar absorption coefficient ε.

From the analysis of absorption features (absorption spectra in solvent with different polarity, such as DMSO and chlorobenzene, were also recorded), some interesting behaviours can be observed, strictly depending on the position of the donor groups on the DBF structure. In fact, going from **TG1** to **TG3** (as well as from **TG2** to **TG4**) the lowest-energy absorption peak undergoes a slight perturbation, since the excitation in both dyes is associated to a single particle transition between orbitals essentially localized on the DBF core and the π-conjugated bridge. Moreover, in **TG3** and **TG4** an additional peak appears at about 350 nm, which can be attributed to π−π* transitions localized on the diphenylamine and the DBF core, as suggested by TD-DFT calculations and the low influence of solvent polarity. When comparing **TG1**/**TG3** and **TG2**/**TG4** with **TK2** and **TK3**, respectively, the situation is different. **TK2** and **TK3** differ from **TG2** and **TG4** for the position of diphenylamino groups on the DBF structure. For **TK2** and **TK3** the lowest-energy peak has a marked charge-transfer character, with the HOMO localized mainly on the diphenylamino substituents and the LUMO localized mainly on the cyanoacrylic group and the linker. Therefore, this peak is red shifted with respect to that of **TG1**/**TG3** and **TG2**/**TG4.** Furthermore, the peak at about 350 nm results reduced in **TK2** and **TK3**, with respect to **TG3** and **TG4**, since the excitation associated to a single particle transition is no more localized only on the π system of diphenylamines and DBF. These optical features are a proof of a better coupling between diphenylamines and DBF core for 3,6-functionalized dyes with respect to 2,7-functionalized ones, so that the electron donating capacity of donors is enhanced as well as the electron delocalization in **TK2** and **TK3** with respect to **TG3** and **TG4**. Moreover, the effect of the bridge length on the absorption properties of dyes also turns out to be noteworthy. Comparing the spectra of **TG1**, **TG2**, **TG3** and **TG4**, a red shift in the absorption is observed with the introduction of an additional thiophene ring in the bridge, so that the effective conjugation length is increased in **TG2** and **TG4** with respect to **TG1** and **TG3**. Conversely, **TK2** and **TK3** dyes showed opposite behaviour, since despite a longer conjugated bridge with two thienyl rings, the latter absorbs at higher energies compared to the former. This is because the effective conjugation length is mainly affected by the functionalization in 3,6-positions, while an additional thiophene ring substantially does not impact on the global effective conjugation, but on the contrary, it appears to negatively affect it. HOMO and LUMO energy levels of dyes were estimated by cyclic voltammetry and the results highlight they are suitable to be used as sensitizers in DSSC devices. DSSC solar cells were then fabricated with the dyes **TG1**–**4** and **TK2**–**3**, and the photovoltaic parameters are summarized in Table 4.

The photovoltaic performance of dyes is consistent with the optical and electrochemical properties. **TG1** and **TG2** show very poor efficiencies (1.23% and 2.07%, respectively), as expected, due to the lack of donor groups. **TG3** and **TG4** show enhanced efficiencies, with values of 3.91% and 2.68%, respectively, thanks to the introduction of donor groups in 2,7-positions. On the other hand, **TK2** and **TK3** with donor groups in 3,6-positions are the dyes showing the higher efficiencies, with values of 5.01% and 5.42%, respectively. These results suggest the functionalization of 3,6-positions of DBF molecule as more favourable to achieve higher photovoltaic performance in this class of organic dyes.

**Figure 9:**
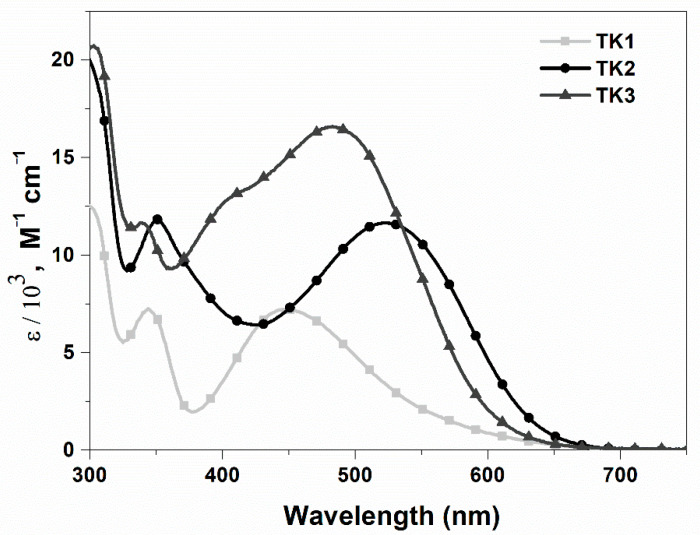
Absorption spectra of **TK1**, **TK2** and **TK3** in CH₂Cl₂ solution. Reprinted (adapted) with permission from ref. [75]. Copyright {2014} “The Royal Society of Chemistry”.

In order to pull out the best photovoltaic potential from this class of DBF-based dyes, our investigation continued focusing the attention on three structures, labelled as **TK1**, **TK2** and **TK3**, characterized by diphenylamine donor groups linked to the 3,6-positions of DBF core and different for the number of thiophene rings between the DBF and the electron-acceptor group (cyanoacrylic): no thiophene ring in **TK1**, one thiophene ring in **TK2** and two thiophene rings in **TK3**. The absorption spectra of such dyes in CH₂Cl₂ solution are shown in Figure 9.

**TK1** shows the maximum absorption peak at 449 nm (ε = 7.3 × 10^3^). The introduction of one thiophene ring as a spacer between the DBF structure and the cyanoacrylic acceptor unit yields the dye **TK2**, which shows a bathochromic shift of 73 nm of the absorption maximum and an increase in the molar extinction coefficient (ε = 1.2 × 10⁴). The introduction of two thiophene rings yields the dye **TK3**, which shows a lower bathochromic shift of 36 nm of the absorption maximum and a higher increase in the molar extinction coefficient (ε = 1.7 × 10⁴). HOMO and LUMO energy levels were estimated by cyclic voltammetry and the data are reported in Table 5, together with those of absorption.

The absorption and electrochemical data indicate the dyes are suitable for application as sensitizers in DSSC solar cells; then, they were used to fabricate photovoltaic devices, in order to investigate their performance. Chenodeoxycolic acid was also used as a co-adsorbent to prevent recombination phenomena which lead to the detriment of cell efficiency. The photovoltaic parameters are summarized in Table 6.

When switching from **TK1** to **TK2** to **TK3** the power conversion efficiency increases from 2.1% to 5.0% to 5.4%, respectively. This is the result of an increase mainly of Jsc, which goes from 4.52 mA cm^−2^ (**TK1**) to 10.82 mA cm^−2^ (**TK2**) to 11.55 mA cm^−2^ (**TK3**), respectively. The IPCE spectra partially explains what happens: the DSSC based on **TK1** shows a lower photoresponse with a maximum of about 20% at 430 nm, compared with the value of about 50% in the region 450–550 nm obtained with the DSSCs based on **TK2** and **TK3**, according with the absorption spectra. Moreover, regarding the dye loading, **TK1** shows a lower adsorption amount compared to **TK2** and **TK3**, probably due to the bulkier molecular structure (the addition of CDCA leads to lower efficiencies), providing a reason for the lower efficiency, assigned to the increased probability of detrimental charge recombination processes. So, the addition of CDCA causes a detrimental effect on the photovoltaic efficiency. To more deeply investigate the relationship between molecular structure and photovoltaic performance, the capacitance, the charge transfer resistance at the TiO_2_/dye/electrolyte interface (Rct) and electron lifetime were measured by electrochemical impedance spectroscopy. For the **TK1**-based DSSC a lower Rct was found, indicating the injected electrons easily undergo to recombination processes. The introduction of one or two thiophene rings in the structure of the dyes (**TK2** and **TK3**, respectively) allows to extend the π-bridge, favouring an increase in the interfacial resistance (also confirmed by the higher Voc value), suggesting a larger number of photo-generate electrons can reach the contact and justifying the higher photocurrent. Furthermore, **TK1** shows a shorter apparent electron lifetime, to confirm an easier recombination of injected electron from **TK1**. Taking into account the planarity of dyes **TK1**–**3**, favoured by the planar core of DBF, a fast intramolecular charge transfer as well as an unfavourable π−π stacking are expected, which may lead to recombination processes. So, the use of CDAC as a coadsorbent is expected to be useful to improve the cell performance, since it can be able to minimize the detrimental dye aggregation by competing with the dye in binding the TiO_2_ surface. DSSCs built by using CDCA as a co-adsorbent yielded 1.1%, 4.72% and 7.45% efficiencies for **TK1**, **TK2** and **TK3** dyes, respectively, highlighting a marked improvement for **TK3**. In the case of **TK1**, the addition of CDAC lowers the efficiency of device, since the only effect of the coadsorbent results in an unfavourable competition with the sensitizer in binding the TiO_2_ surface, without the benefit of minimizing the dye aggregation, since the high steric hindrance of **TK1** directly acts to prevent aggregation processes. The results achieved in this work [75] highlights simple modifications in the molecular structure of a dye, such as the introduction of one or two thiophene rings, can have a strong impact on the chemico-physical properties and on the performance of photovoltaic devices, improving the efficiency from 2.14% to 7.45%.

Continuing further with our interest in deepening the knowledge of the correlation between structure and chemico-physical properties, aimed to improving the photovoltaic performance of DBF-based dyes, we focused our attention on the enhancement of the electron donor power of the diphenylamine units used as donor systems and on the modulation of the π-bridge of the 2D-π-A structure. For this purpose, we synthesized the dyes **TK4**, **TK5** and **TK6**, which can be considered as deriving from the best performing dyes **TK2** and **TK3**, by introduction of para-alkoxy substituted diphenylamines as donor units. The strategy was devoted to structures to pursue characteristics such as high electron-donating ability, reduced dye aggregation and charge recombination, aimed to enhance photovoltaic performances.

**Figure 10:**
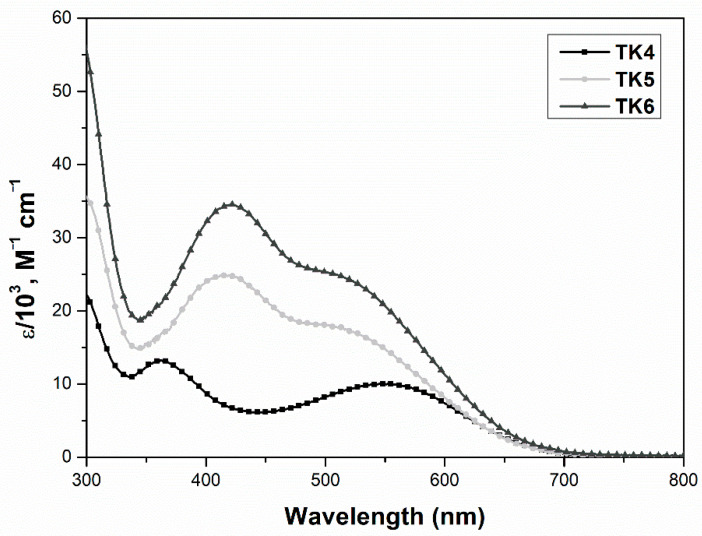
Absorption spectra of **TK4**, **TK5** and **TK6** in CH_2_Cl_2_. Reprinted (adapted) with permission from ref. [104]. Copyright {2016} “Elsevier”.

Photophysical properties were investigated by recording absorption spectra of **TK4**, **TK5** and **TK6** dyes in CH_2_Cl_2_ solution, as reported in Figure 10.

All dyes show strong absorption bands in the ranges 260–350 nm and 350–600 nm, where the higher energy bands are attributed to local π−π* transitions, while the lower energy ones are attributed to charge transfer (CT) transitions. **TK4** shows two maxima peaks at 360 and 548 nm, with extinction coefficients ε = 13,200 and 10,100, respectively. **TK5**, which differs from **TK4** only for the presence of two thiophene rings, instead of one, between the DBF core and the acceptor unit, shows two maxima peaks at 413 and 513 nm, with extinction coefficients ε = 24,800 and 17,800, respectively. **TK6**, which differs from **TK5** for the octyloxy groups, instead of the methoxy groups on the diphenylamine units, shows two maxima peaks at 420 and 516 nm, with extinction coefficient ε = 34,500 and 25,100, respectively. The HOMO energy level was calculated by cyclic voltammetry and the LUMO energy level was derived by the formula E_HOMO_–E_0-0_, were the last is the optical band gap estimated from the absorption edge. The data are reported in Table 7.

The electrochemical data allow us to consider the dyes suitable to be used as sensitizers in DSSCs, so they were tested in liquid cells, and their photovoltaic parameters and performances are reported in Table 8.

Different solvents were tested, reaching the best performance using THF for **TK5** and **TK6**, and CH_3_CN/CHCl_3_ (1/0.01 *v/v*) for **TK4**. Different amounts of CDCA were also tested, reaching the best results with a concentration 15 mM for **TK4** and **TK5** and 5 mM for **TK6**. The reduced amount of CDCA needed for **TK6** is consistent with the structure of such a dye, whose long octyl chains, as expected, act as hindrance to the aggregation of the molecules, lowering the amount of CDCA needed to gain the best photovoltaic performance. Starting from **TK4**, whose maximum efficiency was found to be 5.9%, the addition of a thienyl ring in the π-bridge of the molecular structure leads to **TK5**, which shows a significant improvement of performance with an efficiency of 7.5%. The additional structural variation by long octyloxy chains in **TK6** results in a further increase in photovoltaic performance, reaching a maximum efficiency of 7.8%, together a reduced amount of CDCA needed in the device. The enhancement of photovoltaic efficiencies is mainly the result of an increase in the circuit current Jsc, which value goes from 13.29 mA/cm^2^ for **TK4** to 17.85 and 17.19 mA/cm^2^ for **TK5** and **TK6**, respectively. The behaviour of dyes is confirmed also by the IPCE spectra, in which the **TK4**-based device shows the lower photoresponse, with a maximum of about 55% at 450–550 nm, compared to the plateau of about 70% and 75% in the same region obtained with **TK5**- and **TK6**-based devices, respectively. Interestingly, comparing the photovoltaic parameters of the dyes **TK4** and **TK5** with those of their analogous without methoxy-groups **TK2** and **TK3**, the improvement of the optical properties of the new dyes positively affects the efficiency of the devices. The introduction of the methoxy groups determines a red shift and a widening of the absorption spectra of **TK4** and **TK5**, so their IPCE curves result higher and extended as well as higher result their photocurrent densities. In fact, the Jsc value goes from 10.85 and 14.98 mA/cm^2^ for **TK2** and **TK3**, to 13.29 and 17.85 mA/cm^2^ for **TK4** and **TK5**, respectively, probably for the enhanced light harvesting capability. The results highlight as a simple structural variation consisting in the introduction of alkoxy groups onto the donor units, providing a fine synthetic tool to modulate photovoltaic parameters and enhance the efficienciy of device.

During our ongoing investigations, we finally became interested in studying the effects of a combined functionalization by alkoxy groups onto the donor units and alkyl chains onto the π-bridge, on the optical and photovoltaic properties of DBF-based organic dyes for DSSC. In detail, we focused our attention on the dye **TK3**, one of the best performing in terms of photovoltaic efficiency, using it as starting molecular structure to get new derivatives by functionalization with methoxy or octyloxy groups of the diphenylamine donor units, and with n-hexyl chains of one of the two thienyl rings connecting the DBF core to the acceptor unit. Three new dyes were then obtained, **TK7**, **TK8** and **TK9**, where **TK7** can be considered as deriving from **TK3** by a simple introduction of a n-hexyl chain on the thienyl ring closer the acceptor unit, **TK8** as a further evolution of **TK7** by the introduction of methoxy chains in the para position of diphenylamines, and **TK9** as a further evolution of **TK7** by the introduction of octyloxy chains in the para positions of diphenylamines.

**Figure 11:**
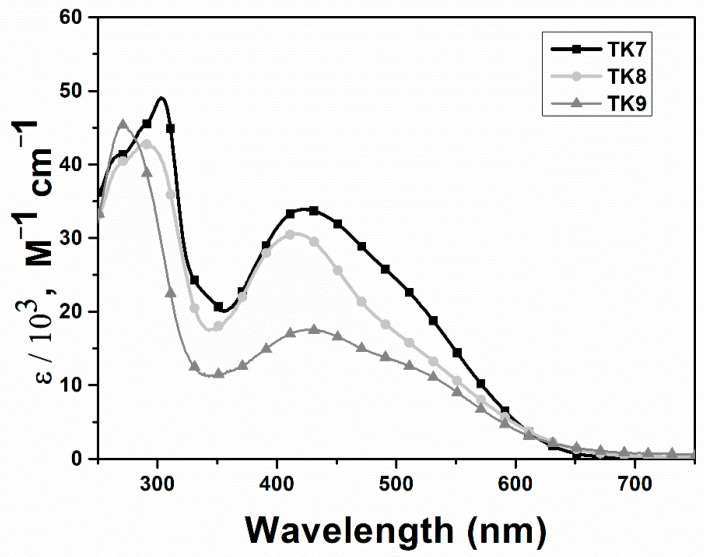
Absorption spectra of **TK7**, **TK8** and **TK9** in CH_2_Cl_2_. Reprinted (adapted) with permission from ref. [103]. Copyright {2016} Elsevier.

The optical properties of **TK7**, **TK8** and **TK9** were investigated by recording absorption spectra in CH_2_Cl_2_ solution (Figure 11); electrochemical properties were recovered by cyclic voltammetry and the data were collected in Table 9.

The dyes show two bands, one at longer wavelengths, in the range 360–600 nm, which is mainly attributed to the charge transfer (CT) from diarylamine donors to cyanoacrylic acid acceptor, and the other in the region 270–350 nm, originating from DBF and thiophene units, which can be attributed to π−π* transitions.

**TK7** shows two absorption maxima at 304 and 422 nm, with extinction coefficient ε = 49,000 and 33,900, respectively. **TK8** and **TK9**, characterized by methoxy and octyloxy groups, respectively, on the diphenylamines, show a reduced absorption capability with respect to **TK7**. **TK8** shows two absorption maxima at 293 and 416 nm, with extinction coefficient ε = 42,500 and 30,600, respectively. **TK9** shows two absorption maxima at 270 and 428 nm, with extinction coefficient ε = 45,180 and 17,500, respectively. By cyclic voltammetry was estimated the HOMO energy level of **TK7**, **TK8** and **TK9**, which was found to be 0.98, 0.94 and 0.93 V, respectively, while the energy gap (E_0-0_) was estimated by the onset wavelength of the absorption spectra, and found to be 2.02, 1.98 and 2.00 eV, respectively. The LUMO energy level was then calculated as the difference HOMO − E_0-0_ and found to be −1.04, −1.04 and −1.07 V (vs NHE), respectively. The optical and electrochemical data prove the dyes are suitable as sensitizer in DSSCs, so their photovoltaic potential was tested in such devices, and the parameters are collected in Table 10.

**TK7** shows the higher efficiency, 7.88%, mainly due to the higher photocurrent density (Jsc 17.82 mA/cm^2^). **TK8** and **TK9** show efficiencies of 6.35% and 6.14%, respectively. The higher value of Jsc in the **TK7**-based device is related to its higher molar extinction coefficient. The IPCEs spectra recorded for the DSSCs are substantially in accordance with the optical properties of the dyes and in agreement with the higher light-harvesting capability of **TK7**, whose spectrum shows a plateau at ~80% between 400 and 550 nm, considerably wider with respect to the spectrum observed for **TK8** and higher with respect to the response obtained for **TK9**. Correlating the optical and photovoltaic properties with the structural characteristics, the role of the hexyl chain inserted on the thienyl ring of the π-bridge can be highlighted by comparing the photovoltaic performance of **TK7** and its homologous without the hexyl chain **TK3**, where the maximum efficiency in the former (η 7.88%) is reached with a lower amount of co-adsorber CDCA (5 mM), with respect to the latter (η 7.45%, CDCA 10 mM). In the comparison between dyes, results show that the alkoxy groups (in **TK8** and **TK9**) on the diphenylamine donor units negatively impact photovoltaic performance, suggesting that in such a branched dye structure, although they space the molecules preventing undesirable dye aggregation, on the other side they can lead to a less-uniform coverage of the TiO_2_ surface.

## 3. Conclusions

In the present review, we have reported some of our contributions in the field of functional organic materials for photovoltaic applications. We have highlighted the synthetic aspects inherent to the synthetic methodologies exploited for their preparation, the photophysical properties and the performance in photovoltaic devices. Organometallic methodologies were mainly used as synthetic protocols, which allow for a rigorous stereo-control of reaction, then of the stereochemistry of the final products. These protocols are characterized by mild reaction conditions and tolerate a wide variety of functional groups, resulting in key routes to access to a widespread variety of organic semiconductor materials. Afterwards, we have described the optical and electrochemical properties of the synthesized materials and their performance in a device. Finally, we investigated the correlation between chemical structure and properties, highlighting the conjugated structure and the functionalization, understood as a type and position of a functional group onto the conjugated skeleton, which can be used as key tools to finely tune properties, both at a molecular level and in solid state, and then, performance in technological applications. Today, power conversion efficiency, stability and cost are the main open challenges in the OPV technology. In recent years, tremendous strides have been made in all three fields, and it seems reasonable to think the progress will continue in the near future. The development of new materials and the improvement of the understanding of the key physical processes governing the device operation are the two areas where the progress has been most significant. Power conversion efficiency near 20% has been reached in the best OPVs and it is reasonable to expect improvements for the years to come, taking into account the infinite number of potential materials offering endless possibilities and the enhancement in device building. On the other hand, machine learning is a field of computer science with high potential and is very promising for organic photovoltaics research [106,107]. Machine learning methods exploiting their data-analysis capability have the potential to guide researchers to discover and develop new high-performance materials [108,109]. Since the OPV technology is form-free, compatible with large-scale printing methods, requires low-cost production investments, is economically and environmentally sustainable, and although the performance of device is not still comparable with the inorganic counterparts, its implementation in “niche” applications such as building integrated environments or wearable electronics can be very competitive and commercially attractive. Furthermore, the Internet of Things (IoT) is a rapidly developing sector that requires cost-effective, low-energy and maintenance-free power sources for use in offices, human wellness and retail. Photovoltaic technologies, especially OPVs, are interesting energy-harvesting systems for IoT, thanks to their potential for low-power output and portability in miniaturized and grid-independent applications. The OPVs have the potential to be one piece of the puzzle of the future’s energy landscape.

## Figures and Tables

**Table 1:** Photovoltaic performance of BHJ solar cells of **PDTBTFV** and **PDTBTV** in blend with PCBM: open circuit voltage Voc; short circuit current Isc; fill factor FF and power conversion efficiency η.

Polymer	Polymer/PCBM Weight Ratio	Voc [V]	Isc [mA cm^−2^]	FF [%]	η [%]
	2:7	0.73	2.94	38.57	0.83
PDTBTFV	3:7	0.75	3.55	37.97	1.05
	1:1	0.83	4.32	34.56	1.24
	2:7	0.55	3.33	28.83	0.53
PDTBTV	3:7	0.56	2.42	24.50	0.33
	1:1	0.43	3.15	33.51	0.46

Reprinted (adapted) with permission from ref. [31]. Copyright {2014} The Royal Society of Chemistry.

**Table 2:** Electrochemical data, absorption maxima and molar extinction coefficients.

Dye	λmax (nm) ^a^	ε (M^−1^ cm^−1^) ^b^	HOMO (V) (vs. NHE)	LUMO (V) (vs. NHE)	Eg (eV)
**ODy1**	453	14,190	1.22	−1.16	2.38
**ODy2**	454/372	42,130/60,890	0.85	−1.36	2.21
**ODy3**	420/320	69,800/108,120	0.75	−1.75	2.50

^a^ Absorption maximum of dyes in CHCl₃ with conc. ca. 2 μM. ^b^ ε molar extinction coefficient at λmax. HOMO calculated by CV in CH₂Cl₂, with tetrabutylammonium hexafluorophosphate (0.1 M) as the supporting electrolyte, Ag/AgCl as the reference electrode, and Pt as the counter electrode. Scanning rate: 100 mV s^−1^. LUMO was calculated as the difference HOMO—Eg. Eg was estimated from the absorption spectral onsets of the dyes. Reprinted (adapted) with permission from ref. [82]. Copyright {2016} American Chemical Society.

**Table 3:** Photovoltaic parameters for DSSCs based on **ODy1, ODy2** and **ODy3** dyes.

Dye	CDCA (mM)	PCE (%)	Voc (V)	Jsc (mA/cm^2^)	FF	Dye Loading (mol/cm^2^)
**ODy1**	0	4.20	0.704	9.18	0.65	2.9 × 10^−7^
	10	5.16	0.735	10.33	0.68	2.4 × 10^−7^
**ODy2**	0	5.76	0.736	11.50	0.68	3.0 × 10^−7^
	10	6.25	0.765	11.85	0.69	2.4 × 10^−7^
**ODy3**	0	4.54	0.689	9.56	0.69	2.8 × 10^−7^
	10	5.00	0.719	9.67	0.72	2.2 × 10^−7^

Reprinted (adapted) with permission from ref. [82]. Copyright {2016} American Chemical Society.

**Table 4:** Photovoltaic parameters for DSSCs based on dyes **TG1**–**4** and **TK2**–**3**.

Dye	η (%)	Voc (V)	Jsc (mA/cm^2^)	FF
**TG1**	1.23	0.56	3.09	0.71
**TG2**	2.07	0.61	5.22	0.65
**TG3**	3.91	0.73	7.50	0.71
**TG4**	2.68	0.65	5.37	0.77
**TK2**	5.01	0.66	10.85	0.70
**TK3**	5.42	0.67	11.55	0.70

Reprinted (adapted) with permission from ref [105]. Copyright {2017} Clearance Center’s RightsLink of Spring Nature.

**Table 5:** Absorption and electrochemical data of **TK1**, **TK2** and **TK3**.

Dye	λ_abs_ (nm) ᵃ	ε × 10^3^ (M^−1^ cm^−1^) ᵃ	HOMO/LUMO (eV)	E_0−0_ ^b^	E_ox_^c^ (V)
**TK1**	449	7.3	−5.35/−3.17	2.18	1.29
**TK2**	522	12	−5.08/−3.13	1.96	1.03
**TK3**	485	17	−4.99/−2.91	2.08	0.94

^a^ Absorption maximum measured in CH_2_Cl_2_. ^b^ Bandgap estimated from the onset of absorption spectrum. ^c^ Oxidation potential in CH_2_Cl_2_ solution containing 0.1 M of NBu_4_PF_6_ with a scan rate of 100 mV s^−1^ (vs. NHE) and calibrated against ferrocene. Reprinted (adapted) with permission from ref. [75]. Copyright {2014} “The Royal Society of Chemistry”.

**Table 6:** Photovoltaic parameters for DSSCs based on dyes **TK1**, **TK2** and **TK3**.

Dye	CDCA ᵃ (mM)	η (%)	FF	Voc (V)	Jsc (mA cm^−2^)	Dye Loading (10^−7^ mol cm^−2^)
**TK1**	0	2.14	0.75	0.63	4.52	2.1
	10	1.08	0.70	0.70	2.21	1.5
**TK2**	0	5.01	0.70	0.66	10.85	2.5
	10	4.72	0.73	0.73	8.85	1.8
**TK3**	0	5.42	0.70	0.67	11.55	2.6
	10	7.45	0.71	0.70	14.98	2.0
**N719**	0	8.11	0.70	0.79	14.68	1.7

^a^ CDCA = chenodeoxycholic acid. Reprinted (adapted) with permission from ref. [75]. Copyright {2014} The Royal Society of Chemistry.

**Table 7:** Absorption and electrochemical data of **TK4**, **TK5** and **TK6**.

Dye	λ_abs_ (ε × 10^3^ M^−1^ cm^−1^) ^a^ (nm)	HOMO ^b^	E_0-0_^c^	LUMO ^d^
**TK4**	548 (10.1)	0.64	1.82	−1.18
**TK5**	513 (17.8)	0.68	1.90	−1.22
**TK6**	516 (25.1)	0.73	1.88	−1.15

^a^ Absorption maximum in CH_2_Cl_2_ (10^−5^ M) solution. ^b^ HOMO (vs. NHE) of the dyes by cyclic voltammetry in CH_2_Cl_2_ solution containing 0.1 M of tetrabutylammonium hexafluorophosphate, with a scan rate of 100 mV^−1^ and calibrated against ferrocene. ^c^ The bandgap, E_0-0_ was determined from the onset of absorption spectrum. ^d^ LUMO = HOMO − E_0-0_. Reprinted (adapted) with permission from ref. [104]. Copyright {2016} “Elsevier”.

**Table 8:** Photovoltaic performance of **TK4**, **TK5** and **TK6**.

Dye	η (%)	FF	Voc	Jsc	Dye Loading ^a^
**TK4**	5.9	0.67	0.667	13.29	2.0
**TK5**	7.5	0.64	0.653	17.85	1.9
**TK6**	7.8	0.69	0.663	17.19	1.6

^a^ [1 × 10^−7^ mol_*_cm^−2^]. Reprinted (adapted) with permission from ref. [104]. Copyright {2016} Elsevier.

**Table 9:** Absorption and electrochemical data of **TK7**, **TK8** and **TK9**.

Dye	λmax (nm) ^a^ (ε × 10^3^/M^−1^ cm^−1^)	HOMO (V) ^b^ (vs. NHE)	LUMO (V) ^c^ (vs. NHE)	E_0−0_^d^ (eV)
**TK7**	422 (33.9)	0.98	−1.04	2.02
**TK8**	416 (30.6)	0.94	−1.04	1.98
**TK9**	428 (17.5)	0.93	−1.07	2.00

^a^ Absorption maximum in CH_2_Cl_2_ (10^−5^ M) solution. ^b^ HOMO of dyes by cyclic voltammetry in 0.1 M TBAPF_6_ in CH_2_Cl_2_ solutions as supporting electrolyte, Ag/AgCl as the reference electrode, and Pt as the counter electrode, and calibrated against ferrocene. Scanning rate: 100 mV s^−1^. ^c^ LUMO was calculated by HOMO−E_0-0_. ^d^ E_0-0_ was estimated from the onset of the absorption spectrum. Reprinted (adapted) with permission from ref. [103]. Copyright {2016} Elsevier.

**Table 10:** Photovoltaic parameters of **TK7**, **TK8** and **TK9** in DSSC devices.

Dye	η %	FF	Voc (V)	Jsc (mA/cm^2^)	Dye Loading (10^−7^ mol/cm^2^)
**TK7**	7.88	0.66	0.67	17.82	2.3
**TK8**	6.35	0.67	0.66	14.33	2.1
**TK9**	6.14	0.67	0.68	13.48	1.7

Reprinted (adapted) with permission from ref. [103]. Copyright {2016} Elsevier.
